# Shared digital music streaming and parent–adolescent communication: evidence from Turkish Cypriot adolescents

**DOI:** 10.3389/fpsyg.2026.1948330

**Published:** 2026-10-07

**Authors:** Ozgur Tosun

**Affiliations:** Department of Communication and Media, Eastern Mediterranean University, Famagusta, Türkiye

**Keywords:** adolescent music listening, digital music streaming, intergenerational communication, parent–adolescent communication, Spotify, Turkish Cypriot adolescents, Uses and Gratifications Theory

## Abstract

**Background:**

The rapid expansion of online music streaming platforms has transformed adolescents’ everyday media practices, making music consumption increasingly individualized, algorithmically curated, and socially networked. While existing scholarship has established the importance of music in adolescent identity formation and emotional regulation, considerably less attention has been devoted to understanding how digitally mediated music listening influences communication within families. As digital-native adolescents increasingly consume music through platforms such as Spotify and YouTube, parents—many of whom remain digital immigrants—face new opportunities and challenges in maintaining meaningful communication with their children. This study investigates whether shared digital music listening constitutes a communicative practice capable of strengthening parent–adolescent relationships. Rather than examining music consumption solely as an individual behavior, the study conceptualizes shared streaming experiences as an interactional process through which emotional exchange, cultural identity, and intergenerational understanding are negotiated.

**Methods:**

Drawing upon a mixed-methods dataset comprising 500 Turkish Cypriot adolescents aged 14–17 (400 quantitative; 100 qualitative), the study integrates responses from a 22-item Music Listening Habits Questionnaire, the Parent–Adolescent Communication Scale (PACS), and qualitative relational content analysis. Pearson correlation analysis was employed to examine associations between shared music listening practices and communication quality, while qualitative coding explored participants’ perceptions of family music experiences.

**Results:**

Quantitative findings demonstrate a statistically significant moderate positive relationship between the frequency of shared music listening with parents and parent–adolescent communication quality (*r* = 0.455, *p* < 0.01). Qualitative evidence further indicates that shared listening to culturally meaningful Turkish Cypriot music facilitates emotional openness, mutual understanding, and constructive family conversations. Although adolescents predominantly accessed music through Spotify and YouTube, the communicative value of music depended less on platform choice than on opportunities for shared engagement and discussion.

**Conclusion:**

The study advances Uses and Gratifications Theory by extending its application from individual media gratification toward shared family media practices. It proposes that digital music streaming can function as an intergenerational communication resource capable of narrowing the communicative divide between digital-native adolescents and digital-immigrant parents. By integrating media studies, communication research, and music psychology, the article offers a culturally grounded framework for understanding how shared digital listening contributes to healthier family relationships in contemporary media environments.

## 1 Introduction

Music has become one of the most pervasive forms of everyday media consumption among adolescents. Unlike previous generations, contemporary teenagers rarely encounter music through broadcast radio or physical recordings alone (Roberts et al., 2009). Instead, music is accessed through an ecosystem of digital platforms that provide continuous, personalized, and on-demand listening experiences (Webster, 2021). Streaming services such as Spotify, YouTube, and Apple Music have fundamentally reshaped how music is discovered, organized, recommended, and shared, transforming music consumption from an activity constrained by ownership into one characterized by permanent connectivity and algorithmic personalization (Barata and Coelho, 2021). This transition has altered not only patterns of media use but also the social environments within which musical experiences are embedded. The global growth of music streaming has accelerated this transformation. Streaming platforms now function simultaneously as entertainment services, recommendation systems, and social networking environments where playlists, listening histories, and musical preferences become visible expressions of identity (Hesmondhalgh, 2025). Algorithmic recommendation engines increasingly influence adolescents’ musical exposure by continuously adapting content to users’ previous listening behaviors, emotional preferences, and engagement patterns. Consequently, musical discovery has become less dependent on family members, traditional media gatekeepers, or peer recommendations and more dependent on computational systems that personalize media consumption (Ahmed and El Sheikh, 2025). This shift represents a significant change in the communicative ecology surrounding adolescent music listening, raising important questions regarding how algorithmically mediated listening influences family interaction and intergenerational communication.

The emergence of algorithmic listening coincides with broader generational differences in digital media engagement. Adolescents born into highly connected digital environments are frequently described as digital natives because digital technologies constitute an integral component of their socialization, identity development, and everyday communication practices (Bolduc and Kinnally, 2018). Their parents, by contrast, often adopted digital technologies later in life and therefore approach online media from substantially different experiential and cultural perspectives. These asymmetries in technological familiarity may influence not only media preferences but also the ways family members communicate about digital experiences, negotiate cultural tastes, and interpret online content. Music streaming platforms therefore occupy an important position at the intersection of technological change and family communication, serving simultaneously as spaces of personal autonomy and potential sites of intergenerational interaction (Bonneville-Roussy et al., 2013). Family communication scholars have consistently demonstrated that adolescence represents a developmental period characterized by increasing autonomy, identity exploration, and renegotiation of parent–child relationships (Keijsers and Poulin, 2013). During these years, communication patterns frequently undergo substantial transformation as adolescents seek greater independence while parents attempt to maintain emotional closeness and guidance. Although disagreements concerning values, lifestyles, and media preferences have long formed part of adolescent development, digital media have introduced additional layers of complexity by creating individualized media environments that often remain inaccessible to parents. Headphone listening, personalized playlists, and algorithmically generated recommendations may reduce opportunities for spontaneous family interaction that previously occurred around shared television viewing, radio listening, or collective music experiences (Krause and North, 2014).

At the same time, digital media do not inevitably diminish family communication. Emerging scholarship on family media practices suggests that shared engagement with digital technologies can create new opportunities for interpersonal interaction, emotional disclosure, and collaborative meaning-making (Maihasni et al., 2025). Music occupies a particularly distinctive position within this context because it simultaneously functions as entertainment, emotional expression, identity performance, memory construction, and cultural transmission. Conversations initiated by favorite songs, shared playlists, music videos, or culturally significant musical traditions may provide relatively non-threatening contexts through which adolescents and parents negotiate beliefs, values, and emotional experiences that might otherwise remain difficult to discuss directly (Bosacki and O’Neill, 2013). Despite growing scholarly interest in digital media and family communication, an important gap remains within the literature. Existing research has extensively examined adolescents’ music preferences, identity formation, emotional regulation, peer relationships, and the technological evolution of streaming platforms. Parallel research has investigated parent–adolescent communication, family cohesion, and media socialization (Brown and Larson, 2009). However, comparatively few empirical studies have integrated these research traditions by examining whether shared digital music listening itself functions as a communicative mechanism capable of strengthening parent–adolescent relationships. Even fewer investigations have considered this relationship within culturally specific contexts where music serves not only as entertainment but also as a vehicle for preserving collective identity and intergenerational cultural continuity (Liu et al., 2024).

The Turkish Cypriot context provides a particularly valuable setting for addressing this gap. Adolescents in Northern Cyprus participate in globally networked digital music cultures while simultaneously remaining embedded within local musical traditions that continue to shape family life and cultural identity. This coexistence of global streaming platforms and locally meaningful musical practices offers an opportunity to examine how digital technologies interact with culturally rooted communication patterns. Rather than positioning global and local musical cultures as competing influences, the present study explores how shared engagement with both contemporary streaming platforms and culturally significant music contributes to family communication (Way et al., 2024). The present article builds upon empirical evidence obtained from a mixed-methods investigation involving 500 Turkish Cypriot adolescents aged between 14 and 17 years. The original study combined quantitative survey data with qualitative accounts of adolescents’ music listening experiences to examine relationships between music listening habits and parent–adolescent communication. Quantitative analysis identified a statistically significant moderate positive association between the frequency of shared music listening with parents and communication quality (*r* = 0.455, *p* < 0.01), while qualitative findings demonstrated that listening to Turkish Cypriot music together frequently facilitated emotionally supportive family interactions. Rather than reproducing those findings, the present article reinterprets them through the contemporary lens of digital music streaming and family media practices, thereby extending their theoretical significance beyond the original thesis.

The study makes three principal contributions. First, it reconceptualizes digital music streaming as an interactional communication practice rather than merely an individual media behavior. Second, it extends Uses and Gratifications Theory by demonstrating that media gratifications associated with music may emerge collectively through shared family experiences rather than exclusively through individual consumption. Third, it contributes empirical evidence from a non-Western cultural context that remains comparatively underrepresented within scholarship on digital media, adolescent communication, and family relationships. Collectively, these contributions advance understanding of how everyday digital media practices can foster meaningful intergenerational communication despite widening technological differences between adolescents and their parents. Accordingly, the objectives of this article are threefold: (1) to examine the relationship between adolescents’ shared digital music listening practices and parent–adolescent communication quality; (2) to explore how online music streaming platforms mediate intergenerational communication within Turkish Cypriot families; and (3) to evaluate the usefulness of Uses and Gratifications Theory for explaining shared digital music listening as a contemporary family communication practice.

## 2 Background

### 2.1 Digital music streaming and the transformation of everyday music consumption

The transition from physical music ownership to digital access represents one of the most significant developments in contemporary media consumption. Advances in internet infrastructure, smartphone adoption, and cloud-based technologies have transformed music from a product purchased through physical or digital downloads into a continuously accessible service. Music streaming platforms such as Spotify, YouTube, Apple Music, Deezer, and YouTube Music have fundamentally altered how individuals discover, organize, consume, and share music (Hagen, 2015). Rather than relying on personal collections or broadcast media, listeners increasingly engage with algorithmically curated catalogues that provide immediate access to millions of songs across virtually every genre. Streaming services have introduced a new communicative ecology in which music listening extends beyond private consumption. Playlist sharing, collaborative playlists, recommendation systems, artist-following, and social media integration have transformed music into an interactive and networked practice. Spotify, for example, allows users to construct public identities through playlists, display listening histories, and share musical preferences across multiple social networking platforms. Likewise, YouTube combines audiovisual content, recommendation algorithms, and interactive comment spaces that facilitate both musical discovery and social engagement. Consequently, digital music platforms function simultaneously as entertainment services, communication environments, and identity performance spaces.

Music therefore becomes both a cultural product and a communicative resource through which interpersonal relationships are established and maintained. These developments suggest that contemporary music streaming should not be conceptualized solely as an individual media behavior but as a socially embedded practice with implications extending into family relationships, peer networks, and intergenerational communication. Although considerable attention has been devoted to consumer behavior, recommendation algorithms, and digital music markets, relatively few studies have examined how streaming platforms influence communication within families. Existing research remains predominantly concerned with technological adoption, user satisfaction, and platform economics, leaving important questions regarding interpersonal communication insufficiently explored. This omission is particularly significant given that music has historically functioned as a medium through which families share memories, values, emotions, and cultural traditions. This should not be taken as overstatement; as will be mentioned later (in section 2.5), intergenerational continuity and generational distinction have been strongly linked since the mid-1900s in the context of Western popular music, a nexus that the present study returns to in its interpretation of findings.

### 2.2 Adolescents, music listening, and identity formation

Music occupies a distinctive position within adolescent development because it simultaneously satisfies emotional, psychological, and social needs. Adolescence represents a developmental stage characterized by identity exploration, increasing autonomy, emotional volatility, and expanding peer relationships. During this period, musical preferences frequently become highly salient components of self-definition, allowing adolescents to communicate values, affiliations, aspirations, and emotional experiences that may otherwise remain difficult to articulate verbally (Setjiawan et al., 2022). Developmental psychology consistently demonstrates that identity formation involves experimentation with multiple symbolic resources, including fashion, language, social networks, and media consumption. Music functions as one of the most accessible and emotionally meaningful of these resources because it enables adolescents to express individuality while simultaneously fostering a sense of belonging to broader social groups. Frith (1981) famously described musical taste as a “badge” of identity, emphasizing that preferences communicate social positioning as much as aesthetic appreciation. Contemporary scholarship continues to support this perspective by demonstrating that adolescents frequently employ musical preferences to negotiate peer acceptance, construct personal narratives, and distinguish themselves from parental authority.

The emotional functions of music are equally significant. Music facilitates mood regulation, stress reduction, emotional validation, and cognitive coping during periods of developmental transition (Liu, 2025). Adolescents often select particular songs to manage anxiety, reinforce positive emotions, or process complex interpersonal experiences. DeNora (2016) argues that music operates as a technology of the self through which individuals actively regulate emotional states and organize everyday experiences. Rather than functioning passively, music becomes an active psychological resource that supports emotional resilience and self-reflection (Chen, 2023). Digital streaming technologies have amplified these developmental functions by providing adolescents with unprecedented autonomy over music selection (Campbell et al., 2007). Personalized playlists, unlimited catalogues, and algorithmic recommendations enable young listeners to construct highly individualized musical environments that continuously evolve alongside their identities. Smartphones further facilitate uninterrupted access to music throughout daily activities, making listening an almost constant companion during study, travel, recreation, and social interaction.

However, increased personalization also raises important questions regarding family relationships. Existing scholarship has primarily emphasized music’s influence on peer relationships, identity construction, and emotional development. Comparatively fewer investigations have explored how adolescents’ increasingly individualized music consumption affects communication within families (Loureiro et al., 2024). This imbalance suggests the need for greater integration between developmental psychology, communication studies, and digital media scholarship.

### 2.3 Parent–adolescent communication in contemporary family media environments

Parent–adolescent communication constitutes one of the most extensively studied dimensions of family functioning because it influences psychological adjustment, emotional well-being, behavioral development, and social competence. Effective communication enables adolescents to express concerns, negotiate independence, and receive emotional support while allowing parents to provide guidance during a period characterized by substantial developmental change (Barnes and Olson, 1985). Communication during adolescence differs fundamentally from communication during childhood. As adolescents pursue greater autonomy, family interactions increasingly involve negotiation rather than direct parental authority. Differences in opinions, values, lifestyles, and media preferences often become more pronounced, creating opportunities for both conflict and relational growth (Delsing et al., 2008). The quality of communication therefore depends less on the absence of disagreement than on families’ ability to engage constructively with differing perspectives.

Digital media have substantially altered this communicative landscape. Contemporary families negotiate relationships within environments saturated by smartphones, social networking platforms, streaming services, online gaming, and algorithmically personalized content (Maihasni et al., 2025). These technologies simultaneously create new opportunities for connection and new forms of communicative fragmentation. Adolescents increasingly consume media individually, often within personalized digital ecosystems that differ substantially from those of their parents. Despite concerns regarding digital isolation, recent communication research suggests that technology itself does not determine communication quality. Rather, the social practices surrounding technology use shape relational outcomes (Grajek et al., 2026). Families who engage collaboratively with digital media often report stronger emotional connections than families in which technology functions exclusively as an individual activity. Shared media experiences—including co-viewing films, collaborative gaming, and collective music listening—may therefore function as contexts for conversation, emotional disclosure, and relationship maintenance.

Music is particularly well suited to facilitate such interactions because songs frequently evoke autobiographical memories, cultural values, and emotional experiences that stimulate conversation across generations (Belfi and Jakubowski, 2021). Discussions concerning lyrics, artists, personal memories, or musical traditions may provide accessible entry points into broader conversations regarding identity, relationships, aspirations, and life experiences. Consequently, music listening may function not merely as entertainment but as a communicative practice capable of strengthening family cohesion. Nevertheless, empirical evidence directly examining this relationship remains limited. Much of the existing literature investigates family communication independently of music consumption, while music research frequently prioritizes peer relationships over family interaction. Bridging these fields represents an important opportunity for communication scholarship.

### 2.4 Shared media experiences and music as a communicative resource

Shared media experiences have emerged as an important area within communication research because they illustrate how media consumption extends beyond individual interpretation into collective meaning-making. Family members rarely consume media in isolation from relational contexts; instead, conversations before, during, and after media use frequently shape interpretation, emotional response, and interpersonal understanding. Research on co-viewing television, collaborative gaming, and family internet use demonstrates that shared media engagement contributes to relationship quality by creating opportunities for discussion, humour, emotional support, and shared memory construction. Similar mechanisms appear applicable to music listening. Unlike many forms of entertainment, music often stimulates autobiographical reflection and emotional disclosure, making it particularly effective in facilitating interpersonal communication (Belfi et al., 2016).

Harwood and Wallace (2021) argue that shared musical engagement strengthens interpersonal relationships because musical participation requires coordinated attention, emotional synchronization, and mutual responsiveness. Music functions simultaneously as symbolic communication and shared emotional experience, enabling participants to connect without relying exclusively on verbal interaction (Vidas et al., 2024). This communicative capacity extends across developmental stages, from lullabies in infancy to collaborative listening practices during adolescence and adulthood (Groarke and Hogan, 2018). Within families, shared music listening may encourage discussions regarding cultural heritage, personal experiences, social values, and emotional challenges. Songs frequently become prompts for storytelling, reminiscence, humour, or advice, thereby supporting communication beyond the immediate listening experience. Such interactions may be particularly valuable during adolescence, when direct conversations regarding emotions or identity can become increasingly difficult.

Digital streaming platforms introduce additional dimensions to shared listening. Collaborative playlists, music recommendations, shared subscriptions, and online playlist exchanges enable families to participate in musical interaction despite individualized listening habits. Rather than eliminating collective experiences, streaming technologies may provide alternative mechanisms through which families negotiate musical preferences and communicate across generational differences (Brito and Vieira, 2022). Despite these possibilities, empirical investigation into shared digital music listening remains comparatively underdeveloped. Existing scholarship has focused largely on technological features or consumer behavior rather than communication processes (Hargreaves et al., 2005). Greater attention to shared listening as an interpersonal practice therefore represents an important direction for future research.

### 2.5 Intergenerational communication, digital generations, and music

The distinction between digital natives and digital immigrants has become an influential framework for understanding generational differences in technology use. As defined in the Introduction, this technology familiarity divide (Bolduc and Kinnally, 2018) influences not only media choices but the manner in which families co-engage with digital experiences, while music is an especially compelling area of interest.

Music provides a particularly interesting context for examining these dynamics because musical preferences often reflect generational experiences (Liu, 2024). Parents frequently maintain emotional attachment to music associated with their own adolescence, whereas contemporary adolescents engage with rapidly evolving digital music cultures shaped by streaming platforms and algorithmic recommendations. These differing musical worlds may either reinforce generational distance or create opportunities for meaningful dialogue when families engage with one another’s musical preferences. Intergenerational communication through music has received increasing scholarly attention. Research demonstrates that shared musical experiences contribute to emotional bonding, empathy, and mutual understanding across generations (Boer and Abubakar, 2014). Musical exchange allows parents and adolescents to share personal histories, cultural traditions, and emotional experiences while simultaneously recognizing differences in identity and taste. Within digitally mediated environments, these interactions become increasingly significant because streaming platforms expose families to unprecedented musical diversity. Adolescents may introduce parents to contemporary artists, while parents may share culturally significant or historically meaningful music. Such reciprocal exchanges transform music from an object of consumption into a mechanism for intergenerational learning and communication.

It must be recognized that the link between music and generation is also historically specific, and is perhaps complicated even more by these shifts in patterns of digital consumption. At least from the 1950s on, when rock and roll began to emerge as a genre of popular music in the West, popular music has been inextricably linked to ideas of youth and inter-generational difference, often to the exclusion of similar links to the preceding generations (Frith, 1981). This relationship between music and generationally defined taste groups seems to be relaxing, at least in part, in modern consumption of digital music. This may be due to the recent ‘retro’ waves that continue to push music across age cohorts (Reynolds, 2011), the increasing number of ‘omnivorous’ listeners who listen across genre and across era (Johnson, 2018), and platforms’ boosting of mood-based and situation-based listening styles, which aspire to supplant the genre- and era-based segmentation of listener tastes that has historically defined generational taste. This does not negate the core argument of this study, which is that shared listening can facilitate intergenerational communication; rather, it raises the question that streaming is not just a second resource for youths and families connecting with their pre-existing music cultures. From a more encompassing point of view, the communicative account should not be seen as an opposition but as a nuance to the sense of stark generational divisions being made by digital platforms in the field of culture more generally. The patterns outlined throughout this section of parental digital access and technological fluency are also varied by socio-economic and geographic location; as with the digital-native/digital-immigrant rubric, these should not be interpreted as representing a continuum of digital access across generations.

### 2.6 Turkish Cypriot cultural context

Communication practices cannot be fully understood independently of their cultural environments. Northern Cyprus provides a particularly valuable context for examining relationships between music, digital media, and family communication because local musical traditions coexist alongside intensive engagement with global streaming platforms. Adolescents simultaneously participate in internationally connected digital music cultures while remaining embedded within families that maintain strong cultural identities rooted in Turkish Cypriot history, language, and musical heritage. Traditional Turkish Cypriot music continues to occupy an important place within family celebrations, community gatherings, religious ceremonies, and cultural festivals (Sadikoglu, 2021). These musical practices function not merely as entertainment but as mechanisms through which collective memory, cultural identity, and intergenerational continuity are maintained. Consequently, music frequently carries symbolic meanings extending beyond aesthetic appreciation.

The coexistence of local traditions and global streaming services creates distinctive communicative dynamics. Adolescents access international musical repertoires through Spotify and YouTube while also encountering locally meaningful music within family settings (Aguiar and Waldfogel, 2021). Rather than replacing traditional musical practices, digital platforms increasingly facilitate access to both global and local musical content. This dual exposure creates opportunities for intergenerational dialogue regarding cultural identity, heritage, and changing media practices. Evidence from the underlying mixed-methods investigation demonstrates that shared listening to Turkish Cypriot music frequently generated positive family conversations and emotional closeness among participants, suggesting that culturally meaningful music retains important communicative functions even within highly digitalized media environments.

### 2.7 Research gap

In light of the gap identified in the Introduction, two additional limitations specific to the contribution of the present study need to be highlighted. Furthermore, existing studies remain disproportionately concentrated in Western European and North American contexts, limiting understanding of how culturally embedded musical traditions interact with contemporary streaming technologies in other societies (American Academy of Child and Adolescent Psychiatry, 2020). Research specifically examining Turkish Cypriot adolescents remains extremely limited despite the coexistence of strong local musical traditions and widespread adoption of global streaming platforms. Finally, although Uses and Gratifications Theory has frequently been employed to explain individual motivations for music consumption, considerably less attention has been devoted to understanding how media gratifications emerge collectively through shared family experiences. Addressing these gaps requires an integrated theoretical perspective that combines digital media scholarship, communication studies, developmental psychology, and cultural analysis. The present study responds to this need by examining shared digital music listening as an intergenerational communication practice situated within the cultural context of Turkish Cypriot families.

### 2.8 Theoretical framework

#### 2.8.1 Uses and Gratifications Theory

Uses and Gratifications Theory (UGT) provides the principal theoretical foundation for the present study because it conceptualizes media audiences as active participants who intentionally select media to satisfy psychological, emotional, cognitive, and social needs (Bhatiasevi, 2024). Rather than viewing audiences as passive recipients of media effects, UGT argues that individuals actively choose particular media according to the gratifications they seek. Media consumption therefore reflects purposeful behavior shaped by personal motivations, situational circumstances, and anticipated outcomes.

Although originally developed within the context of traditional mass communication, UGT has demonstrated remarkable adaptability across successive waves of technological innovation, including the internet, social media, smartphones, streaming platforms, and algorithmically personalized digital environments. Contemporary applications increasingly recognize that media users actively construct individualized consumption patterns by selecting platforms, content, and interaction styles that correspond to evolving psychological and social needs (Ahmad and Burke, 2025).

Within music research, UGT has consistently demonstrated that adolescents consume music for multiple interconnected reasons, including emotional regulation, entertainment, identity expression, social affiliation, relaxation, cognitive stimulation, and companionship. Digital streaming services expand these gratifications by enabling immediate access to personally meaningful music regardless of time or location (İnanç, 2026).

##### 2.8.1.1 Media choice in algorithmically curated environments

Traditional UGT assumes that audiences consciously select media to satisfy particular needs. Contemporary streaming platforms complicate this assumption because recommendation algorithms increasingly shape media exposure. Spotify, YouTube, and similar platforms continuously personalize recommendations according to previous listening behaviors, creating dynamic interactions between user agency and computational curation. The notion of platform affordance allows for a closer examination of adolescent choices such as platform evaluation, playlist building, song skipping, following artists, and music shares as both an enabler and a disabler rather than independent of the platform that enables such actions. The framing is promoted with evidence of the involvement of algorithmic and editorial logics in the editorial process of platforms, non-algorithmically (Bonini and Gandini, 2019) acting. In this definition, media choice does not solely come from the free will of the people or simply a set of pre-programmed algorithmic determinisms, but from the affordance structures/prioritizations that take on the media platforms themselves and make some things easy, visible, or assumed and others more difficult, less visible, or less presumed.

##### 2.8.1.2 Emotional gratification through shared listening

Music has long been recognized as an effective resource for emotional regulation. Yet emotional gratification frequently extends beyond individual listening into shared interpersonal experiences (American Academy of Pediatrics, 2009). Families who listen to music together may experience emotional synchronization, empathy, nostalgia, and collective memory construction. Such shared gratifications support relationship maintenance by creating emotionally meaningful opportunities for conversation and mutual understanding (Bakagiannis and Tarrant, 2006). The present study therefore conceptualizes emotional gratification as simultaneously individual and relational, arguing that shared listening produces communicative benefits unavailable through solitary consumption alone.

##### 2.8.1.3 Identity formation through digital music practices

UGT also provides a useful framework for understanding music as a resource for identity construction. Adolescents actively employ musical preferences to communicate personal values, social affiliations, and cultural identities. Digital streaming platforms amplify this process by allowing users to curate public playlists, share favorite artists, and construct visible musical identities within online environments. Importantly, identity formation occurs not only within peer relationships but also through interaction with parents. Discussions concerning musical preferences, cultural traditions, and generational differences enable adolescents to negotiate autonomy while maintaining family relationships. Music therefore functions simultaneously as an expression of individuality and a medium for interpersonal communication.

##### 2.8.1.4 Family communication as a shared media gratification

The present study proposes that communication itself constitutes an important gratification emerging through collaborative media engagement. Shared digital music listening enables families to exchange memories, discuss values, negotiate identities, and strengthen emotional bonds (Baym and Ledbetter, 2009). Communication therefore becomes not merely a consequence of media use but one of the gratifications actively sought through shared musical experiences. This reconceptualization positions music streaming as a family communication resource rather than solely an entertainment technology. Accordingly, the theoretical model underpinning this article proposes that adolescents’ motivations for digital music consumption extend beyond individual enjoyment to include opportunities for emotional connection, identity negotiation, and intergenerational communication. Through this expanded interpretation, Uses and Gratifications Theory provides an integrative framework linking media choice, emotional gratification, identity formation, and family communication within contemporary digital media environments.

## 3 Method

### 3.1 Research design

This study employed a convergent mixed-methods research design to investigate the relationship between adolescents’ digital music listening practices and parent–adolescent communication. The mixed-methods approach was selected because the research sought not only to determine whether statistically significant relationships existed between music listening behaviors and communication quality but also to understand how adolescents themselves interpreted these experiences within their family environments (Belcher and Haridakis, 2013).

The quantitative component examined the association between specific music listening behaviors and parent–adolescent communication using standardized survey instruments and inferential statistical analysis. The qualitative component complemented these findings by exploring adolescents’ subjective experiences, perceptions, and meanings attached to shared music listening with their parents. Integrating both approaches enabled statistical relationships to be interpreted within the broader social, cultural, and communicative contexts in which family interactions occur.

Rather than treating the quantitative and qualitative findings as independent investigations, both strands were designed to address the same overarching research objective from complementary perspectives (Fetters and Tajima, 2023). Quantitative analysis established the magnitude and direction of relationships between music listening variables and communication quality, whereas qualitative analysis provided explanatory insight into the communicative processes underlying these statistical associations. This integration strengthened the interpretive validity of the study by combining numerical evidence with participants’ lived experiences.

### 3.2 Study context

The research was conducted in the Turkish Republic of Northern Cyprus, where adolescents experience simultaneous exposure to globally distributed digital music platforms and locally embedded musical traditions. This context provides an appropriate setting for examining how contemporary music streaming practices intersect with culturally rooted family communication. The increasing popularity of Spotify, YouTube, Apple Music, and similar streaming services has substantially altered adolescents’ everyday media practices within Northern Cyprus (Aksoy, 2021). At the same time, Turkish Cypriot music continues to occupy an important place within family life, cultural celebrations, and intergenerational interaction. Consequently, the study offers an opportunity to examine communication processes situated at the intersection of digital globalization and local cultural identity.

### 3.3 Participants and sampling

The study involved a total sample of 500 adolescents between the ages of 14 and 17 years, consistent with the developmental period during which parent–adolescent relationships undergo substantial transformation. The quantitative phase included 400 participants, while the qualitative phase consisted of 100 participants who completed open-ended survey questions regarding their music listening experiences and family communication. Participants were recruited from secondary schools in Northern Cyprus using procedures designed to ensure representation across the target adolescent age group. The study included both male and female students enrolled in different schools, thereby increasing the diversity of the sample and improving the generalizability of the findings within the Turkish Cypriot adolescent population. Demographic characteristics of the participants are presented in the Results section. The selected age range was theoretically appropriate because adolescence represents a developmental period characterized by increasing autonomy, identity exploration, evolving media practices, and changing communication patterns with parents. These developmental characteristics make adolescents particularly suitable for examining the communicative functions of digital music listening.

### 3.4 Data collection instruments

#### 3.4.1 Music listening habits questionnaire

Adolescents’ music listening behaviors were measured using a researcher-developed 22-item Music Listening Habits Questionnaire. The instrument assessed multiple dimensions of music consumption, including listening frequency, preferred music genres, motivations for listening, music sharing behaviors, discussions about music with parents, and music listening through online streaming platforms. Several questionnaire items specifically examined whether adolescents listened to music together with their parents, discussed favorite artists and songs, experienced parental approval or criticism of their musical preferences, and perceived music as facilitating meaningful family conversations. These variables constituted the principal independent variables examined in the quantitative analyses. The questionnaire additionally collected information regarding participants’ preferred digital music platforms, allowing examination of contemporary streaming behaviors within the Turkish Cypriot context.

#### 3.4.2 Parent–adolescent communication scale (PACS)

The quality of family communication was measured using the Parent–Adolescent Communication Scale (PACS) developed by Barnes and Olson (1982). PACS is one of the most widely applied instruments for evaluating communication quality between adolescents and their parents and has demonstrated satisfactory psychometric properties across diverse cultural settings. The instrument evaluates multiple dimensions of communication, including openness, mutual understanding, emotional expression, trust, responsiveness, and perceived effectiveness of family communication. Higher scores indicate more positive communication characterized by openness, reciprocal understanding, and constructive interpersonal interaction. Within the present study, PACS served as the primary dependent variable against which adolescents’ music listening behaviors were statistically examined.

#### 3.4.3 Qualitative survey

To complement the quantitative findings, participants in the qualitative phase completed a structured open-ended questionnaire consisting of 12 qualitative questions. These questions encouraged adolescents to describe their experiences of listening to music with parents, discussing musical preferences, sharing favorite songs, and reflecting upon the influence of music on everyday family communication. The qualitative component enabled participants to explain how shared listening experiences contributed to emotional closeness, conflict resolution, cultural identity, and communication within their households.

#### 3.4.4 Data collection procedure

Following institutional ethical approval, permission was obtained to conduct the research within participating secondary schools. Eligible students received information describing the purpose of the study, voluntary participation procedures, confidentiality protections, and their right to withdraw without consequence. Because participants were minors, the research followed established ethical procedures governing adolescent participation. Data collection was conducted within school environments under appropriate supervision while maintaining participants’ anonymity and confidentiality. Students first completed the quantitative questionnaires, after which participants assigned to the qualitative phase responded to the open-ended survey questions. Data collection was completed during the same research period using standardized administration procedures to ensure consistency across participating schools.

### 3.5 Reliability and measurement quality

The internal consistency of the quantitative instruments was evaluated prior to hypothesis testing. Reliability analysis demonstrated satisfactory internal consistency for both the Music Listening Habits Questionnaire and the Parent–Adolescent Communication Scale, indicating that the instruments were appropriate for subsequent statistical analysis. Assessment of internal consistency was conducted using Cronbach’s alpha, which is widely accepted as an indicator of scale reliability within communication and behavioral research. The reported reliability analyses supported the use of both instruments for measuring adolescents’ music listening behaviors and parent–adolescent communication. The satisfactory reliability of the instruments increases confidence that observed statistical relationships reflect meaningful associations rather than measurement error.

### 3.6 Quantitative data analysis

Quantitative analyses were conducted to examine relationships between adolescents’ music listening behaviors and parent–adolescent communication quality. Initially, descriptive statistics were generated to summarize participants’ demographic characteristics, music listening habits, preferred streaming platforms, and communication scores. Subsequently, Pearson product–moment correlation analysis was employed to determine the strength and direction of associations between music listening variables and parent–adolescent communication. Pearson’s correlation coefficient was selected because the principal research objective involved evaluating linear relationships between continuous variables measured using standardized instruments.

Correlation coefficients were interpreted according to established guidelines for behavioral science research, with statistical significance evaluated at the 0.05 and 0.01 probability levels. Particular emphasis was placed on the relationship between the frequency of shared music listening with parents and communication quality, which constituted the study’s principal hypothesis. Where appropriate, regression analysis was used to visualize the relationship between shared music listening and parent–adolescent communication, thereby facilitating interpretation of the observed statistical association.

### 3.7 Qualitative data analysis

Qualitative responses were analysed using relational content analysis, an approach that enables systematic examination of conceptual relationships among participants’ narratives rather than merely identifying isolated themes.

Data analysis proceeded through multiple iterative stages. Initially, participant responses were reviewed repeatedly to achieve familiarity with the data. Meaningful textual segments were subsequently coded according to recurring concepts relating to music listening, emotional interaction, communication, cultural identity, family relationships, and shared musical experiences. Coding procedures were performed using Atlas.ti, which facilitated systematic organization, comparison, and visualization of qualitative data. Similar codes were progressively grouped into higher-order conceptual categories that represented broader patterns of communication emerging across participants’ responses (Carley, 1993).

Finally, thematic relationships among categories were examined to identify recurring communicative processes linking shared music listening with parent–adolescent interaction. The resulting thematic framework provided explanatory insight into the quantitative findings by illustrating how shared musical experiences contributed to emotional openness, mutual understanding, and culturally meaningful family communication.

### 3.8 Ethical considerations

The study adhered to internationally recognized ethical principles governing research involving human participants. Ethical approval was obtained before commencement of data collection, and all research procedures complied with institutional requirements for studies involving adolescent participants. Participation was entirely voluntary. Students received clear information regarding the objectives of the research, confidentiality procedures, and their right to discontinue participation at any stage without penalty. Personal identifiers were removed from the dataset to preserve anonymity, and all responses were analysed in aggregate form to protect participants’ privacy.

Given the involvement of adolescents between the ages of 14 and 17 years, additional safeguards were implemented to ensure that participation remained ethically appropriate. These procedures included informed consent, confidentiality protection, secure handling of research data, and adherence to established standards for educational and communication research involving minors. The integration of rigorous quantitative procedures with systematic qualitative analysis, together with careful ethical oversight, provides a robust methodological foundation for examining how shared digital music listening contributes to parent–adolescent communication within the Turkish Cypriot context.

## 4 Results

### 4.1 Overview of the findings

This study investigated whether adolescents’ digital music listening practices, particularly shared listening experiences with parents, are associated with the quality of parent–adolescent communication. The mixed-methods design enabled the integration of quantitative evidence obtained from standardized survey instruments with qualitative insights generated through relational content analysis. Collectively, the findings demonstrate that shared music engagement extends beyond entertainment and functions as a meaningful communicative practice within Turkish Cypriot families.

The quantitative findings consistently revealed statistically significant associations between several music-related behaviors and parent–adolescent communication. Among these variables, the frequency of shared music listening with parents exhibited the strongest positive relationship with communication quality. Additional analyses demonstrated that discussing favorite music genres, engaging in meaningful conversations initiated by music, and positive perceptions of music-related interaction were also associated with healthier family communication. Conversely, parental judgement of adolescents’ musical preferences was negatively associated with communication quality.

The qualitative findings complemented these statistical relationships by illustrating the interpersonal processes through which music facilitated emotional closeness, strengthened cultural identity, encouraged mutual understanding, and created opportunities for meaningful family interaction.

### 4.2 Participant characteristics

The study consisted of 500 adolescents between the ages of 14 and 17 years, comprising 400 participants in the quantitative phase and 100 participants in the qualitative phase. The sample represented adolescents enrolled in secondary schools across Northern Cyprus and included both male and female students. The qualitative participants reflected diverse musical preferences while demonstrating widespread adoption of digital music streaming platforms. Spotify emerged as the dominant streaming service among participants, followed by Apple Music, with YouTube also constituting a commonly used platform for music discovery and consumption (Table 1).

**Table 1:** Qualitative participant demographics (*N* = 10).

Participant	Gender	Age	Music preference(s)	Platform preference
R1	Male	16	Rock, metal	Spotify
R2	Male	14	Metal, rock	Spotify
R3	Female	17	Rap, arabesque	Apple Music
R4	Male	15	Arabesque	Spotify
R5	Male	16	Arabesque	Spotify
R6	Female	17	Nostalgic pop, rap	Spotify
R7	Female	14	Alternative rock, rap	Apple Music
R8	Female	16	Turkish rock, metal	Spotify
R9	Male	15	Metal, rock, rap	Spotify
R10	Female	17	Rock, metal	Spotify

R = Respondent. Data derived from qualitative participant interviews.

The demographic composition provided a suitable basis for examining contemporary music listening practices among digitally connected adolescents while capturing variation in age, gender, musical preferences, and streaming platform use.

### 4.3 Digital music streaming preferences

Analysis of participants’ media consumption indicated that online music streaming had become deeply embedded within adolescents’ everyday lives. The majority of qualitative participants reported Spotify as their primary music platform, while Apple Music represented the second most frequently identified subscription service. YouTube continued to serve an important complementary role, particularly for music discovery, live performances, and music video consumption.

Rather than functioning solely as technological tools for accessing music, these platforms represented environments through which adolescents organized playlists, explored new genres, exchanged recommendations with peers, and occasionally shared musical experiences with family members. The findings suggest that the communicative significance of streaming platforms lies not in the platform itself but in the opportunities, they create for interpersonal interaction. Adolescents who reported discussing music with parents, sharing playlists, or listening together described these activities as facilitating conversation rather than replacing face-to-face communication.

These findings support contemporary perspectives that digital streaming platforms increasingly operate as social communication environments rather than merely repositories of musical content.

### 4.4 Shared music listening and parent–adolescent communication

The principal hypothesis examined whether the frequency of shared music listening with parents was associated with the quality of parent–adolescent communication. Pearson product–moment correlation analysis revealed a moderate positive relationship between these variables (r = 0.455, *p* < 0.001), indicating that adolescents who listened to music together with their parents more frequently also reported significantly higher levels of positive communication. This relationship represents the strongest positive association observed among the principal music-related variables investigated in the study (Table 2).

**Table 2:** Pearson correlation between shared music listening with parents and parent–adolescent communication quality.

Variables	Pearson correlation (r)	Significance (p)	Interpretation
Shared music listening with parents ↔ Parent–adolescent communication quality	0.455	< 0.001	Moderate positive relationship

Sample: *N* = 500.

The strength of the correlation indicates that shared listening is more than just for leisure. Similar to this, music may serve as a context that parents and adolescents use to communicate ideas, feelings, and experiences related to better interpersonal understanding, although it is not causal since this is suggested from the cross-sectional design but not proven. The linear regression model further illustrates this relationship (Figure 1).

**Figure 1:**
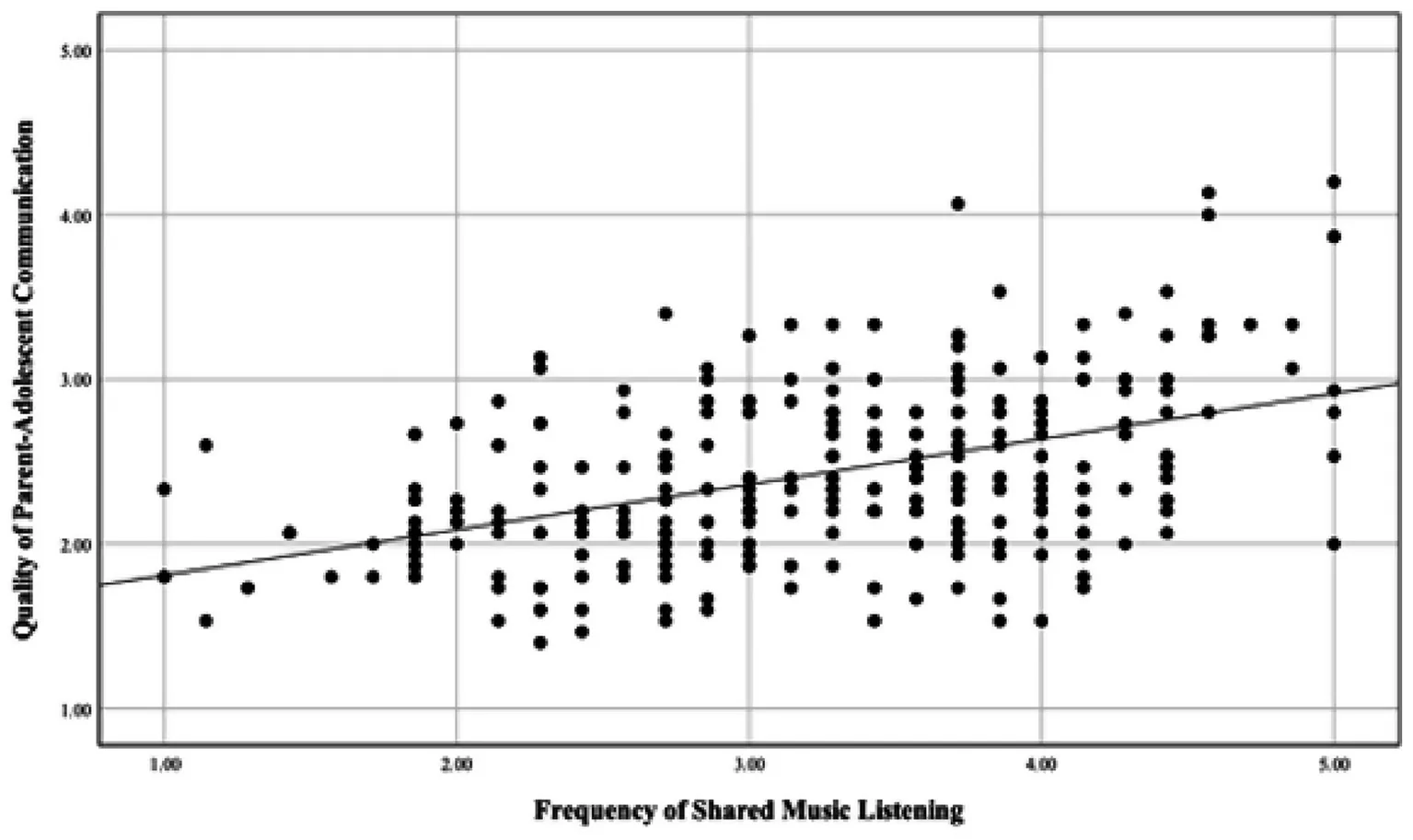
Linear regression of relationship between the frequency of shared music listening and the quality of parent-adolescent communication.

The positive slope of the regression line demonstrates that increased frequency of shared music listening corresponded with progressively higher parent–adolescent communication scores. Although individual variation remained evident, the overall trend clearly indicates that shared musical engagement is associated with healthier communication patterns. From a communication perspective, these findings suggest that music functions as a conversational catalyst rather than merely an entertainment medium. Shared listening experiences appear to reduce communicative barriers by creating naturally occurring opportunities for interaction, emotional expression, and reciprocal engagement.

### 4.5 Discussion of favorite music genres

The study further examined whether conversations regarding favorite music genres were associated with communication quality. Pearson correlation analysis demonstrated a statistically significant positive relationship between discussing favorite music genres and parent–adolescent communication (r = 0.347, *p* < 0.001) (Table 3).

**Table 3:** Pearson correlation between discussing favorite music genres and parent–adolescent communication quality.

Variables	Pearson correlation (r)	Significance (p)	Interpretation
Discussing favorite music genres ↔ Parent–adolescent communication quality	0.347	< 0.001	Moderate positive relationship

Sample: *N* = 500.

Although the association was weaker than that observed for shared listening itself, the findings indicate that conversations surrounding musical preferences contribute positively to family communication. These findings suggest that communication benefits emerge not only from listening together but also from discussing the meanings attached to music. Conversations concerning favorite artists, song lyrics, musical memories, and genre preferences may encourage adolescents to articulate personal experiences while simultaneously enabling parents to gain greater understanding of their children’s identities and emotional worlds. The results therefore indicate that music-related dialogue constitutes an important communicative mechanism within family interaction.

### 4.6 Parental judgement of musical preferences

An important finding concerns adolescents’ perceptions of parental judgement regarding their musical preferences. Pearson correlation analysis identified a statistically significant negative relationship between perceived parental judgement and parent–adolescent communication (r = −0.341, *p* < 0.001) (Table 4).

**Table 4:** Pearson correlation between parental judgement of music preferences and parent–adolescent communication quality.

Variables	Pearson correlation (r)	Significance (p)	Interpretation
Parental judgement of adolescents’ music preferences ↔ Parent–adolescent communication quality	−0.341	< 0.001	Moderate negative relationship

Sample: *N* = 500.

This inverse relationship indicates that adolescents who perceived greater criticism or judgement of their musical tastes tended to report poorer communication with their parents.

The findings suggest that music functions as a symbolic representation of adolescent identity. Consequently, criticism directed toward musical preferences may be interpreted not merely as disagreement concerning entertainment but as rejection of broader aspects of personal identity, autonomy, and self-expression. But this interpretation is not the only one which need be considered. Musical taste and attitude might have something to do with adolescents’ emotional or affective tone, and away from reference to identity the criticism may be experienced as a violation of emotional attunement. Conversely, parents who demonstrate openness toward adolescents’ musical interests may create communicative environments characterized by greater trust, mutual respect, and emotional disclosure.

### 4.7 Meaningful conversations initiated by music

The study also investigated whether music stimulated meaningful conversations between adolescents and their parents. The results demonstrated a moderate positive relationship between the frequency of meaningful conversations arising from music and communication quality (r = 0.444, *p* < 0.001) (Table 5).

**Table 5:** Pearson correlation between meaningful conversations initiated by music and parent–adolescent communication quality.

Variables	Pearson correlation (r)	Significance (p)	Interpretation
Meaningful conversations initiated by music ↔ Parent–adolescent communication quality	0.444	< 0.001	Moderate positive relationship

Sample: *N* = 500.

This finding closely parallels the relationship identified for shared listening frequency and represents one of the strongest associations observed throughout the quantitative analyses.

In this way music was not used as a source of background amusement but as a prompt for wider comment including emotion, relationships, memoirs, beliefs and everyday experiences, consistent within such families as a conversational trigger. Songs therefore operate as conversational resources that facilitate interpersonal interaction within families. These findings reinforce the proposition that communication benefits emerge primarily through interaction surrounding music rather than through passive listening alone.

### 4.8 Positive perceptions of music listening

The final quantitative analysis examined adolescents’ agreement with the statement assessing positive perceptions of music listening in relation to communication. Pearson correlation analysis demonstrated another statistically significant positive association (r = 0.402, *p* < 0.001) (Table 6).

**Table 6:** Pearson correlation between positive perceptions of music listening and parent–adolescent communication quality.

Variables	Pearson correlation (r)	Significance (p)	Interpretation
Positive perceptions of music listening ↔ Parent–adolescent communication quality	0.402	< 0.001	Moderate positive relationship

Sample: *N* = 500.

This result indicates that adolescents who perceived music as positively influencing family relationships also reported higher overall communication quality.

Taken together, the quantitative findings consistently demonstrate that music-related interaction contributes positively to parent–adolescent communication, whereas perceived parental criticism of musical preferences exerts the opposite effect.

### 4.9 Qualitative findings

The qualitative phase provided important explanatory insight into the statistical relationships identified through the quantitative analyses.

Relational content analysis generated five overarching conceptual categories:

- Emotional impact
- Music genres
- Music listening habits
- Perceptions of music listening preferences
- Shared music listening

These categories collectively describe the communicative processes through which music influenced family relationships (Figure 2).

**Figure 2:**
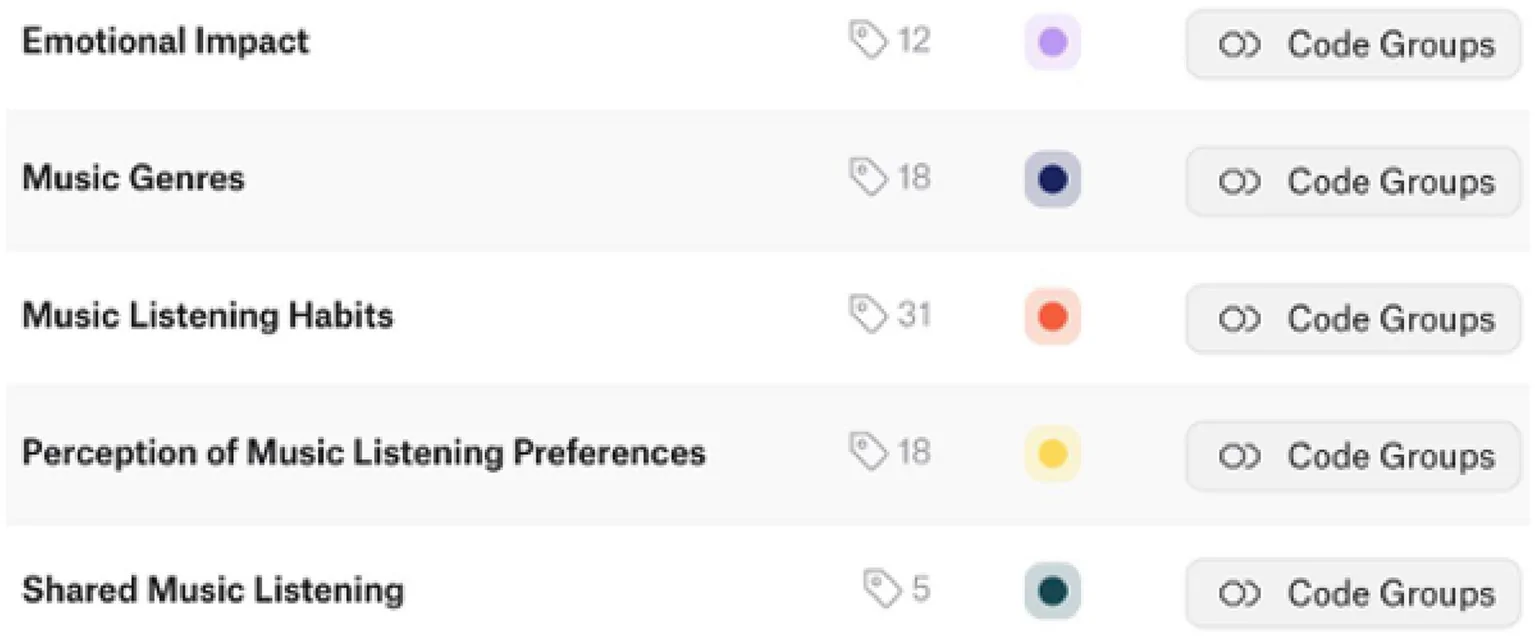
Code groups generated in Atlas.ti.

The distribution of qualitative codes further demonstrated that emotional closeness constituted the most frequently occurring concept, followed by emotional connection, emotional distance, shared experiences, and communication.

The prominence of emotionally oriented codes indicates that participants primarily interpreted music as an interpersonal rather than purely recreational experience (Figure 3).

**Figure 3:**
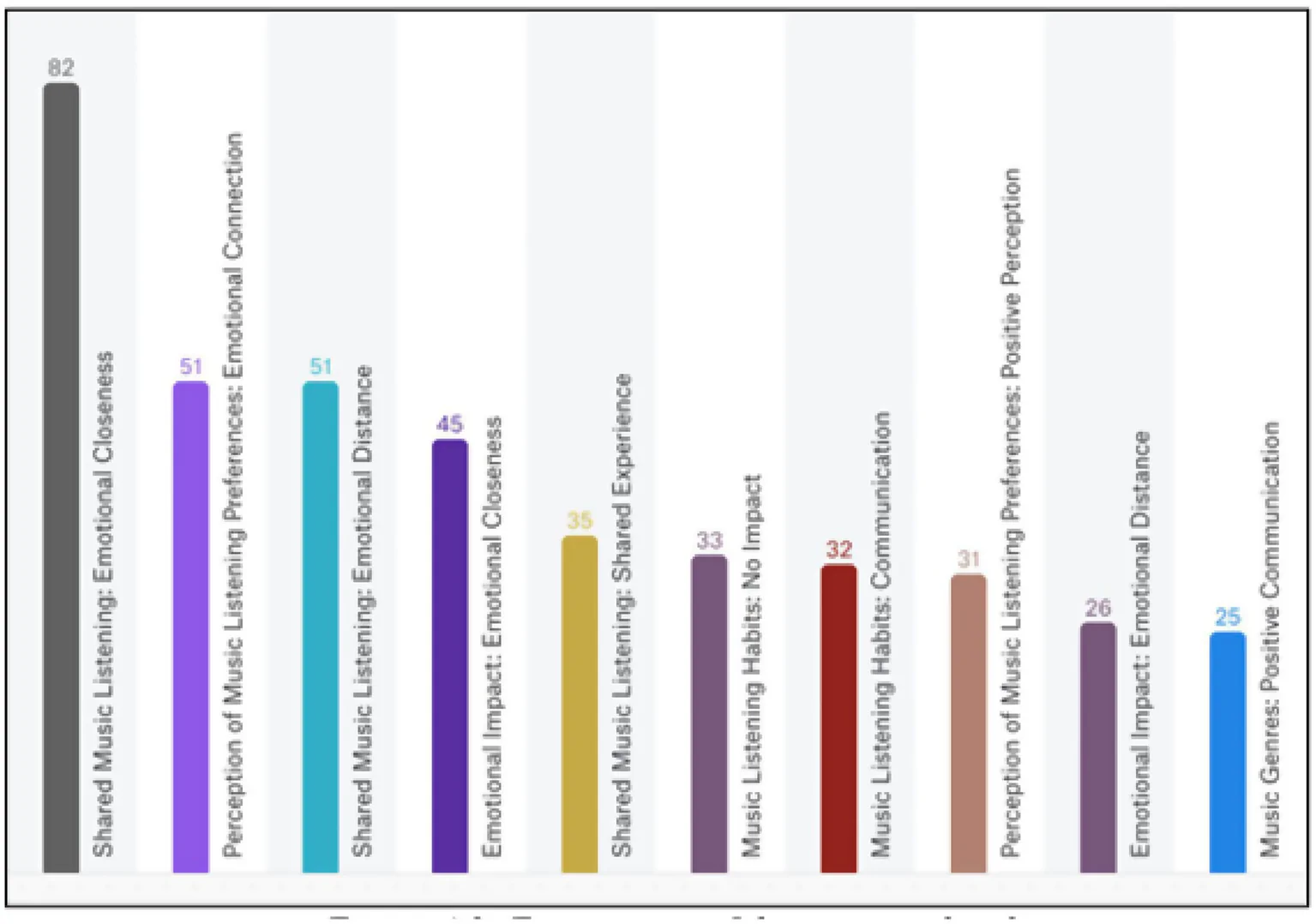
Frequencies of the most used codes.

### 4.10 Thematic patterns emerging from the qualitative analysis

Quantification of qualitative responses revealed several dominant themes.

Within the category of emotional impact, 46.4% of participants associated shared music listening with bonding, whereas 28.6% described music as encouraging discussion. Smaller proportions identified conflict (14.3%) or reported no enhancement of communication (10.7%). Regarding music genres, 45.5% indicated that music promoted positive communication, while 36.3% specifically identified Turkish Cypriot music as facilitating stronger family interaction. Only 14.5% associated music with negative communication, and 3.7% reported no observable impact. Within music listening habits, communication (33.9%) and positive relationships (30.2%) emerged as the dominant themes. Appreciative attitudes toward shared listening accounted for 15.5%, whereas 20.4% perceived little communicative influence. Analysis of music listening preferences indicated that 42.5% of adolescents described emotional connection with parents, compared with 25.8% reporting positive perceptions generally. Emotional disconnection (19.1%) and negative perceptions (12.6%) occurred considerably less frequently.

Finally, within the category of shared music listening, 45.1% of participants described experiences of emotional closeness, 28.1% referred to emotional distance, 19.2% emphasized shared experiences, and only 7.6% reported limited opportunities for shared listening. Collectively, these qualitative findings strongly support the quantitative evidence by demonstrating that music facilitates communication primarily through emotional bonding, reciprocal understanding, shared cultural experiences, and opportunities for meaningful interpersonal interaction.

### 4.11 Integration of quantitative and qualitative findings

The integration of quantitative and qualitative findings demonstrates remarkable convergence across both strands of the study.

Quantitative analyses consistently showed statistically significant positive relationships between shared music practices and communication quality, with shared listening frequency (r = 0.455) and meaningful conversations initiated by music (r = 0.444) representing the strongest associations. Simultaneously, qualitative narratives illustrated the communicative mechanisms underlying these statistical relationships, highlighting emotional closeness, shared cultural identity, mutual appreciation, and increased openness in family conversations.

Importantly, Turkish Cypriot music emerged as a culturally significant resource that strengthened intergenerational dialogue, suggesting that communication benefits arise not solely from listening together but from engaging with music that carries shared cultural meaning.

Overall, the findings support the proposition that digital music streaming platforms acquire communicative significance when they facilitate shared listening experiences rather than exclusively individualized media consumption. Music therefore functions as a relational resource through which adolescents and parents negotiate identity, emotion, and interpersonal understanding within contemporary digitally mediated family environments.

## 5 Discussion

The present study sought to examine whether adolescents’ digital music listening practices, particularly shared listening experiences mediated through contemporary streaming platforms, contribute to the quality of parent–adolescent communication. By integrating quantitative evidence with qualitative accounts from Turkish Cypriot adolescents, the findings demonstrate that music functions as considerably more than a recreational activity. Instead, shared musical engagement emerges as a relational communication practice through which families negotiate emotions, identities, cultural meanings, and interpersonal understanding. This interpretation extends existing scholarship by positioning digital music streaming not merely as a technological innovation but as a communicative environment capable of strengthening intergenerational relationships.

The strongest quantitative finding—the moderate positive relationship between shared music listening and parent–adolescent communication (*r* = 0.455, *p* < 0.001)—provides compelling evidence that regular musical engagement between parents and adolescents is associated with healthier family communication. This relationship should really be restated in a variance form to help determine the interpretative weight of this relationship. The value for r is 0.455 with an r^2^ about 0.207, meaning that when compared to the variance in parent–adolescent communication scores, shared listening frequency accounts for about one-fifth of the variance. The rest is due to factors that aren’t contained within the current model — parenting style, family structure, individual temperament, and all the other factors that impact family communication, as noted under Limitations. It should therefore not be interpreted as proof that parenting quality will significantly improve when families share music, but that music is one of several measured, modifiable factors that can be affecting parenting quality. It should therefore not be regarded as a finding that music is a major determinant of parenting quality, but that it is one of many factors measured and modifiable that can affect parenting quality.

Although correlation does not imply causation, the consistency of the findings across multiple music-related variables suggests that music operates as an important communicative resource within family life. Adolescents who listened to music with their parents more frequently also reported greater openness, emotional closeness, and communication quality. These results were reinforced by qualitative narratives describing shared listening as an opportunity for conversation, bonding, and mutual understanding.

This finding aligns closely with Harwood and Wallace’s (2021) argument that shared musical engagement promotes interpersonal coordination and emotional synchronization. With Harwood and Wallace (2021) propose that collaborative musical experiences facilitate relationship development because participants simultaneously attend to common emotional stimuli while engaging in reciprocal social interaction. The present findings extend this proposition beyond musical performance into the everyday context of digital streaming, demonstrating that shared listening through platforms such as Spotify and YouTube can perform comparable communicative functions. Rather than replacing interpersonal interaction, digital music appears capable of initiating conversations that might otherwise not occur within everyday family routines.

Similarly, the findings support DeNora's (2000) conceptualization of music as a resource for organizing emotional and social life. DeNora argues that individuals actively employ music to regulate emotional states, construct personal identities, and organize everyday experiences. The present study extends this perspective by demonstrating that emotional regulation frequently occurs within relational contexts rather than exclusively through solitary listening. Shared musical experiences enabled adolescents and parents to discuss emotions, memories, aspirations, and everyday concerns in ways that promoted mutual understanding. Music therefore functioned simultaneously as an individual emotional resource and a collective communicative practice.

A similar correlation, although more conceptually distinct, was seen for meaningful conversations by music (r = 0.444, *p* < 0.001), which was the second highest of all of the correlations from the study. It is a variable to which one should assign as much as possible an analytical distinction from a shared listening frequency, since the two represent two different mechanisms. Shared listening frequency is a measure of co-presence at the time the user is listening to music, and meaningful conversations indicates whether the music is heard as a discursive, conversational, event. The identical effect size for both variables suggests that this is a close link in practice; that is, if families are listening it is likely that they will also talk about what they have heard, but the qualitative information (Sections 4.9–4.10) suggests the mechanisms are not the same. A series of qualitative narratives were assigned to the ‘shared experiences’ category where a child and an adult were listening to music together but not having a conversation, and to the ‘discussion’ line of others that were not listening together but were interacting with a child about music they heard elsewhere (e.g., adults talking about music that a child heard through a closed door). For the theoretical claim advanced in Section 2.8.1.4: if adolescents and parents are seeking gratification through communication, then it is the taking up of communication through shared music listening (in addition to its frequency) that is more direct as a behavioral indicator of that seeking. It is difficult without using the cross-sectional design to determine if listening together leads to talk or if talk is simply an overrepresentation of listening, a directionality question that will be emphasized in the longitudinal work proposed in Section 8.

The positive relationship identified between discussing favorite music genres and communication quality further illustrates the communicative significance of music beyond listening itself. Although the observed correlation (*r* = 0.347, *p* < 0.001) was weaker than that associated with shared listening, it nevertheless demonstrates that conversations surrounding musical preferences contribute positively to family relationships. These findings support the study of Schwartz and Fouts (2003) that musical preferences represent symbolic expressions of personal identity. Adolescents frequently communicate values, social affiliations, emotional experiences, and aspirations through their musical choices. Consequently, discussions concerning favorite genres provide parents with opportunities to understand dimensions of adolescent identity that may not emerge through conventional family conversations.

The symbolic-identity framing used thus far needs to be qualified. There are two distinct ways of thinking about musical preference in the parent–adolescent relationship, one as a symbol that serves to communicate aspects of identity, values, and social belonging (as emphasized by Schwartz and Fouts, 2003) and another as the more direct stimulus of resonance or affect (where a song matters for the reason that it feels a certain way rather than meaning something about identity and social affiliation). These channels may be working together in reality, though they are not conclusive as pointers to an identity-signaling account of the phenomenon – but they were not ruled out, and the qualitative theme ‘emotional connection’ (42.5% of coded responses under music listening preferences; Table 7) is equally consistent with an affective-resonance account. Future research aimed at differentiating between these mechanisms, such as catching between adolescents’ reports of ‘this is who I am’ and ‘this is how I feel’, would further strengthen the theoretical claims that have been made here.

**Table 7:** Quantified qualitative themes and frequencies.

Category	Theme	Frequency (%)
Emotional impact	Bonding	46.4
Emotional impact	Discussion	28.6
Emotional impact	Conflict	14.3
Emotional impact	No enhancement of communication	10.7
Music genres	Positive communication	45.5
Music genres	Turkish Cypriot music promotes communication	36.3
Music genres	Negative communication	14.5
Music genres	No observable impact	3.7
Music listening habits	Communication	33.9
Music listening habits	Positive relationships	30.2
Music listening habits	Appreciative attitudes	15.5
Music listening habits	No communicative influence	20.4
Music listening preferences	Emotional connection	42.5
Music listening preferences	Positive perceptions	25.8
Music listening preferences	Emotional disconnection	19.1
Music listening preferences	Negative perceptions	12.6
Shared music listening	Emotional closeness	45.1
Shared music listening	Emotional distance	28.1
Shared music listening	Shared experiences	19.2
Shared music listening	Limited shared listening	7.6

The findings also correspond with Rentfrow et al. (2011) work demonstrating that music serves multiple psychological and social functions throughout adolescence. Their research emphasizes music’s role in mood regulation, social affiliation, and identity formation. The present study complements this literature by illustrating that these functions are not confined to peer relationships. Rather, when parents actively engage with adolescents’ musical interests, music becomes a mechanism through which emotional support and family cohesion are strengthened. This interpretation broadens existing developmental perspectives by recognizing parents as continuing participants in adolescents’ musical worlds despite increasing autonomy during adolescence.

Conversely, the statistically significant negative relationship between parental judgement of musical preferences and communication quality (*r* = −0.341, *p* < 0.001) highlights the importance of parental acceptance within contemporary family communication. Adolescents who perceived greater criticism of their musical preferences reported lower communication quality, suggesting that parental evaluations of music extend beyond aesthetic disagreement. Because musical preferences frequently constitute highly salient components of adolescent identity, criticism directed toward preferred genres or artists may be interpreted as rejection of the adolescent rather than merely rejection of musical taste.

These findings resonate strongly with Ter Bogt et al.'s (2011) research concerning intergenerational transmission of musical preferences. Although parents inevitably influence children’s musical development through socialization, contemporary adolescents increasingly exercise independent control over music selection via personalized streaming services. This autonomy represents an important developmental achievement; however, it may simultaneously create opportunities for conflict when parental preferences differ substantially from those of adolescents. The present study suggests that communication outcomes depend less on agreement regarding musical taste than on parents’ willingness to engage respectfully with adolescents’ musical identities.

A methodological concern that is especially relevant for the finding of positive perceptions of music listening (*p* < 0.001, r = 0.402) is that both the predictor and the outcome of this association was taken from the same adolescent respondent, using the same self-report instrument and during the same administration time. This could lead to shared-method variance (namely young people who, for some reason, tend to have positive views of family life may give higher ratings to both the quality of communication in the family and to the importance of music in family life, regardless of the causal direction). This concern also extends (in smaller proportion) to each of the other correlations presented in Section 4.4–4.8, which are based on a single-informant, single-instrument adolescent self-report (see Section 7). Here the qualitative data provide partial reassurance because they came from open-ended, not scaled, items and converged on the same pattern nonetheless, but as a result of the lack of parent reported and/or observed data the correlations found should be interpreted as association within the domain of adolescents’ subjective experience of family communication, not as an independent behavioral measure of it.

The findings further contribute to scholarship examining music as a facilitator of social identity. Miranda and Claes (2009) argued that music satisfies both individual psychological needs and broader social motivations during adolescence. The present study demonstrates that family communication represents another important social function of music that has received comparatively limited scholarly attention. Adolescents who discussed music with parents, exchanged musical recommendations, or listened together consistently described these experiences as strengthening interpersonal relationships. This is thus a pattern that is consistent with facilitation of identity negotiation within the peer group; but once again, it is not possible to determine the direction from which the facilitation comes in the present design when adolescents are dealing with identity negotiation in a relationship with the parent.

The qualitative findings further enrich this interpretation by identifying emotional closeness, shared cultural identity, positive communication, and parental understanding as dominant themes. Particularly noteworthy is the prominence of Turkish Cypriot music as a catalyst for positive family interaction. More than one-third of participants identified locally meaningful musical traditions as strengthening communication, suggesting that cultural familiarity enhances the communicative functions of music. This observation extends Boer and Abubakar's (2014) cross-cultural findings demonstrating that musical rituals promote cohesion within families. Whereas Boer and Abubakar emphasized music as a socializing practice across collectivist and individualist societies, the present study illustrates how local musical traditions continue to foster intergenerational communication despite adolescents’ extensive engagement with global streaming platforms.

Alongside the quantitative data, there is also a significant minority component that requires specific attention beyond being drawn to within the general themes of the majority data. For the “shared music listening” category, 28.1% of the coded subgroups indicated emotional rapport instead of closeness while for “music listening habits,” 20.4% indicated no communicative influence at all (Table 7). They are too big to consider as noise. When examined with the negative correlation between parent and adolescent satisfaction with shared listening (Section 4.6; r = − 0.341) a reasonable boundary condition becomes apparent: shared listening seems to work as a communicative resource most strongly when it co-occurs with parental openness to the adolescents’ music, and less effectively; or negatively where it fails. Co-presence while listening to music does not alone determine the communicative advantages found elsewhere in the present study, however, there seems to be a moderating effect when listening to music in an accepting or an evaluative manner. This helps to clarify the main argument of the paper: that listening to music is not enough to make families communicate better, but that it does communicate better when listened to without judgement by parents; which can be translated into practical suggestions in Section 6.1.

The coexistence of Spotify, YouTube, and Turkish Cypriot musical traditions also illustrates the increasingly hybrid nature of contemporary media environments. Adolescents no longer experience a dichotomy between global digital culture and local cultural identity. Instead, streaming platforms provide access to both international and culturally specific musical repertoires. Consequently, digital technologies do not inevitably undermine local traditions; rather, they may facilitate renewed engagement with culturally meaningful music when families intentionally participate in shared listening experiences.

Another important contribution concerns the role of algorithmically mediated music consumption within family communication. Contemporary streaming platforms personalize listening through recommendation systems that continually adapt to users’ behaviors. Scholars such as Hagen (2015) and Hagen (2016) have debated whether streaming encourages greater musical diversity or increasingly individualized listening practices. The present findings suggest that algorithmic personalization does not necessarily reduce family communication. Instead, communication outcomes appear to depend on how adolescents and parents incorporate streaming technologies into their everyday relationships. When playlists, recommendations, and musical discoveries become topics of family conversation, streaming platforms facilitate interpersonal interaction rather than social isolation.

From a theoretical perspective, the study significantly extends Uses and Gratifications Theory. It is crucial to clarify the scope of the statements that the available data can provide on this initial theoretical extension. In Section 2.8.1.1, we suggested that the selection of media to be accessed via algorithmic curation is a product of a relationship between adolescent choice and computational personalization. In this study, no measure explicitly operationalized algorithmic exposure, i.e., participants’ algorithms were not measured as a percentage of the listening occasion, nor as an algorithm that selected music that the parent discussed with the participant. The findings in relation to this extension are therefore of an indirect nature: the almost universal dominance among participants of Spotify and YouTube as platforms for sharing listening shows that algorithmic mediated platforms currently dominate the context within which this shared listening occurs (Table 8), although none of them have been tested directly, but rather serve as a reasonable interpretive structure for further analyses. This extension outlined in Section 8 could be assessed separately from a platform preference measure with future research that includes a platform-level listening log.

**Table 8:** Preferred music streaming platforms and music genres (*N* = 10).

Participant	Primary streaming platform	Secondary platform (if applicable)	Preferred music genre(s)
R1	Spotify	—	Rock, metal
R2	Spotify	—	Metal, rock
R3	Apple Music	—	Rap, arabesque
R4	Spotify	—	Arabesque
R5	Spotify	—	Arabesque
R6	Spotify	—	Nostalgic pop, rap
R7	Apple Music	—	Alternative rock, rap
R8	Spotify	—	Turkish rock, metal
R9	Spotify	—	Metal, rock, rap
R10	Spotify	—	Rock, metal

R = Respondent. Data derived from qualitative participant interviews.

Traditional applications of UGT conceptualize music listening primarily as an individual behavior through which audiences satisfy emotional, cognitive, or entertainment needs. The present findings suggest that communication itself constitutes an important gratification emerging through shared media use. Adolescents do not simply consume music to regulate emotions or construct identities; they also employ music to initiate conversations, negotiate family relationships, express cultural belonging, and strengthen interpersonal connections. Gratification therefore extends beyond the individual listener to encompass relational outcomes experienced collectively by families.

This expanded interpretation has broader implications for communication theory. Much contemporary scholarship concerning adolescents and digital media has emphasized technological risks, including social isolation, excessive screen time, and declining face-to-face interaction. While such concerns remain important, the present findings demonstrate that digital technologies can also facilitate meaningful interpersonal relationships when embedded within supportive family practices. The communicative consequences of streaming platforms therefore depend less on technology itself than on the social contexts in which technology is used.

Taken together, the findings position shared digital music listening as an accessible, culturally meaningful, and emotionally engaging practice capable of strengthening communication between adolescents and parents. Rather than viewing streaming technologies as barriers to family interaction, the present study suggests that they can become valuable resources for fostering dialogue, empathy, and intergenerational understanding.

## 6 Practical implications

The findings have important implications for families, educational institutions, mental health professionals, technology developers, and public policy. As digital media become increasingly integrated into adolescents’ everyday lives, understanding how music can facilitate rather than inhibit interpersonal communication becomes increasingly relevant.

### 6.1 Implications for parents

The findings suggest that parents should view adolescents’ musical interests as opportunities for communication rather than sources of conflict. Rather than evaluating unfamiliar genres from a position of criticism, parents may strengthen family relationships by demonstrating curiosity, asking adolescents about favorite artists, discussing song lyrics, and participating in occasional shared listening experiences. The negative relationship between parental judgement and communication quality indicates that acceptance of adolescents’ musical preferences contributes to more open family dialogue. Parents need not share identical musical tastes with their children; however, respectful engagement with adolescents’ musical identities appears substantially more beneficial than dismissing or criticizing those preferences. Simple practices such as collaboratively creating playlists, listening during family travel, or discussing culturally significant songs may create valuable opportunities for emotional interaction.

### 6.2 Implications for schools

Schools represent important environments for promoting healthy family communication alongside academic development. Music education programmes may incorporate activities encouraging students to explore intergenerational musical experiences, interview family members regarding musical memories, or develop collaborative music projects involving parents. Teachers may also integrate discussions concerning music, identity, digital media, and communication into media literacy and citizenship curricula. Such initiatives would encourage students to critically examine how music influences emotions, relationships, and cultural understanding while recognizing the positive communicative potential of digital media.

### 6.3 Implications for counselors and family practitioners

School counselors, psychologists, and family therapists may consider incorporating music into communication-focused interventions involving adolescents and parents. Because music frequently provides emotionally accessible points of discussion, therapists may employ shared musical experiences to facilitate conversations concerning identity, emotional regulation, family conflict, and interpersonal understanding. Music-based interventions may be particularly beneficial during adolescence, when direct communication regarding emotions often becomes challenging. Shared listening activities, lyric discussions, and collaborative playlist development represent practical strategies for strengthening family dialogue within counseling settings.

### 6.4 Implications for music streaming platforms

The findings also carry implications for developers of digital music platforms. Existing streaming services primarily optimize individual user engagement through personalized recommendation algorithms. Future platform development could place greater emphasis on family-oriented features that encourage collaborative listening and intergenerational interaction. Potential innovations include enhanced collaborative family playlists, conversation prompts accompanying culturally significant music, shared listening modes designed for household participation, and recommendation systems that identify music appealing across generations. Such features would position streaming platforms not only as personalized entertainment services but also as technologies supporting family relationships.

### 6.5 Implications for media literacy

Media literacy initiatives frequently emphasize online safety, misinformation, privacy, and critical evaluation of digital content. The present findings suggest that media literacy programmes should additionally recognize music as a form of interpersonal communication. Adolescents and parents alike may benefit from educational programmes exploring how digital music influences identity, emotional well-being, cultural belonging, and family relationships. Developing awareness of music’s communicative functions may encourage more intentional media practices in which streaming platforms become resources for dialogue rather than exclusively individual entertainment.

### 6.6 Implications for family and cultural policy

At the policy level, the findings support initiatives promoting family participation in cultural and musical activities. Community music programmes, local festivals, intergenerational cultural events, and educational campaigns encouraging shared family music experiences may contribute positively to adolescent well-being and family cohesion. Within the Turkish Cypriot context, preserving local musical traditions alongside participation in global digital music cultures represents an important opportunity for strengthening cultural continuity across generations. Policies supporting access to local musical heritage through both traditional and digital platforms may therefore contribute simultaneously to cultural preservation and healthier family communication. Collectively, these practical implications suggest that music should be recognized not merely as entertainment but as an accessible communication resource capable of strengthening interpersonal relationships across families, educational institutions, and wider society.

### 6.7 Limitations

Although the present study contributes important empirical evidence regarding the relationship between adolescents’ digital music listening practices and parent–adolescent communication, several limitations should be acknowledged when interpreting the findings.

First, the study was conducted exclusively within the Turkish Cypriot context. While this cultural setting represents an important contribution to communication research because it remains comparatively underrepresented in studies of digital media and family communication, the cultural specificity of the sample may limit the direct generalizability of the findings to other national, linguistic, or sociocultural environments. Family communication patterns, parenting practices, music consumption behaviors, and cultural meanings attached to music vary considerably across societies. Consequently, the observed relationships should be interpreted within the cultural context in which the research was conducted.

Second, the quantitative component employed a cross-sectional research design. Although statistically significant associations were identified between shared music listening and parent–adolescent communication, cross-sectional data cannot establish causal relationships. It therefore remains unclear whether shared music listening contributes to stronger communication, whether families with healthier communication naturally engage in more shared musical activities, or whether both processes operate simultaneously through reciprocal influence. Longitudinal research would provide greater insight into the temporal dynamics underlying these relationships.

Third, the study relied primarily on adolescents’ self-reported perceptions of their music listening behaviors and communication with parents. Self-report measures are widely used within communication research because they provide direct access to participants’ subjective experiences; however, they remain vulnerable to social desirability bias, recall bias, and individual differences in self-perception. Parents’ perspectives were not collected, preventing direct comparison between adolescent and parental evaluations of shared musical experiences and communication quality.

Fourth, although the study incorporated a mixed-methods design, qualitative data were collected through open-ended survey responses rather than extended interviews or participant observation. While relational content analysis provided valuable insight into participants’ experiences, more intensive qualitative approaches could generate deeper understanding of how communication unfolds during shared listening experiences within everyday family settings.

Fifth, the study examined music listening primarily within the context of widely used digital streaming platforms, including Spotify, YouTube, and Apple Music. Although these services represent dominant forms of contemporary music consumption, digital media environments continue to evolve rapidly. New platforms, recommendation systems, and social media ecosystems increasingly influence adolescents’ music discovery and listening behaviors. Consequently, the findings should be interpreted as reflecting the technological environment existing at the time of data collection rather than representing a fixed pattern of digital music consumption.

Finally, although Uses and Gratifications Theory provided a valuable framework for explaining adolescents’ motivations for music consumption and communication, family relationships are shaped by numerous psychological, developmental, and contextual variables beyond music listening alone. Factors such as parenting style, socioeconomic conditions, family structure, personality, mental health, peer relationships, and broader digital media practices were not examined directly within the present study. Future investigations integrating these variables may provide a more comprehensive understanding of the complex factors influencing parent–adolescent communication. Despite these limitations, the study offers one of the few mixed-methods examinations of shared digital music listening and family communication within the Turkish Cypriot context. The convergence of quantitative and qualitative findings strengthens confidence in the observed patterns while providing an important foundation upon which future research can build.

## 7 Future research directions

The rapid evolution of digital media technologies presents numerous opportunities for extending research on music listening and parent–adolescent communication. Building upon the findings of the present study, several promising directions warrant further investigation.

One important avenue concerns the growing influence of artificial intelligence on music discovery and consumption. Contemporary streaming services increasingly employ sophisticated machine-learning algorithms to personalize recommendations according to users’ listening histories, emotional preferences, contextual behaviors, and engagement patterns. Future research should examine whether AI-driven recommendation systems strengthen or weaken opportunities for intergenerational communication. Comparative studies could investigate whether algorithmically generated recommendations encourage shared listening experiences between parents and adolescents or whether highly personalized recommendation systems contribute to increasingly individualized media environments that reduce family interaction. Relatedly, future investigations should explore the communicative implications of algorithmically curated playlists. Automatically generated playlists such as personalized daily mixes, mood-based recommendations, and discovery playlists have become central components of digital music streaming. Research is needed to determine whether these algorithmic features influence family communication differently from manually created playlists. In particular, studies could compare collaborative family playlists with algorithmically generated playlists to determine which forms of music organization more effectively promote interpersonal interaction and emotional connection.

Another promising direction involves examining the expanding role of short-form video platforms such as TikTok in shaping adolescents’ musical experiences. Increasingly, young people encounter new music through viral videos, user-generated content, influencer recommendations, and algorithmically promoted audio clips rather than through traditional music streaming alone (Avdeeff, 2014). Future studies should investigate how music discovery through TikTok differs from Spotify or YouTube in influencing family communication, cultural identity, and intergenerational understanding. Particular attention should be given to whether music associated with social media trends creates new opportunities for conversation or contributes to widening generational differences in musical preferences. Future research should also investigate collaborative digital music practices within families. Shared playlists, collaborative playlist creation, family music libraries, and co-listening features available through streaming platforms remain relatively unexplored within communication scholarship. Experimental and longitudinal studies could examine whether intentionally designed family playlists improve communication quality, emotional closeness, and mutual understanding over extended periods. Such work would provide valuable evidence concerning whether digital music platforms can be deliberately employed as interventions for strengthening family relationships.

Cross-cultural comparative research represents another important priority. The present study contributes evidence from Turkish Cypriot adolescents; however, music carries different cultural meanings across societies. Comparative investigations involving adolescents from diverse cultural, linguistic, and religious backgrounds would enable researchers to examine whether the communicative functions of shared music listening remain consistent across cultures or whether they vary according to family structures, parenting practices, collective values, and musical traditions. Such comparisons would substantially strengthen the generalizability of theoretical explanations concerning music and intergenerational communication. Longitudinal research designs would further strengthen understanding of developmental processes linking music listening and family communication. Following adolescents across multiple years would allow researchers to determine whether shared music listening influences communication trajectories over time or whether communication quality predicts subsequent musical engagement. Such evidence would provide stronger foundations for causal interpretation than cross-sectional designs alone.

Future studies should additionally incorporate the perspectives of multiple family members. Collecting data from adolescents, mothers, fathers, and siblings would enable researchers to compare perceptions of shared musical experiences within the same household. Multi-informant designs would provide richer understanding of family communication dynamics while reducing reliance on single-source self-report data. Advances in digital behavioral analytics also offer valuable opportunities for future communication research. With appropriate ethical safeguards and informed consent, researchers could combine self-reported perceptions with objective streaming data, playlist histories, listening frequency, and collaborative music-sharing behaviors obtained directly from digital platforms. Such approaches would enable more precise examination of relationships between actual music consumption and family communication.

Finally, future theoretical work should continue expanding Uses and Gratifications Theory beyond individual media consumption toward relational and family-oriented media practices. The present study demonstrates that communication itself may constitute an important gratification emerging through shared digital media experiences. Extending this conceptual framework to other digital media—including podcasts, streaming video platforms, multiplayer games, and emerging immersive media—may provide broader theoretical insight into how contemporary technologies facilitate interpersonal relationships within families. Collectively, these research directions acknowledge that digital media ecosystems continue to evolve at a rapid pace.

## Data Availability

The original contributions presented in the study are included in the article/supplementary material, further inquiries can be directed to the corresponding author.
